# Extracellular Vesicles: A Multidimensional Role in the Occurrence and Development of Nasopharyngeal Carcinoma

**DOI:** 10.3390/biom16020267

**Published:** 2026-02-09

**Authors:** Huining Chen, Hejing Huang, Song Qu

**Affiliations:** Department of Radiation Oncology, Guangxi Medical University Cancer Hospital, Nanning 530021, China; 202520700@sr.gxmu.edu.com (H.C.); 202320711@sr.gxmu.edu.com (H.H.)

**Keywords:** extracellular vesicles, nasopharyngeal carcinoma, tumor microenvironment, biomarkers, therapy

## Abstract

Extracellular vesicles (EVs) have garnered significant attention in cancer research, as they enable the regulation of the occurrence, progression, and metastasis of tumors. This narrative review summarizes studies published between 2020 and 2025 from PubMed, focusing on nasopharyngeal carcinoma and extracellular vesicles. We analyze the function and mechanism of EVs in the tumor microenvironment, biomarkers, and treatment. Numerous studies have attempted to explain the mechanism of NPC-EVs affecting tumor microenvironments through the transmission of its cargo. And liquid-biopsy technology using EVs as biomarkers, such as exosomal cyclophilin A, the phosphatase and tensin homolog, and EVs-miR-30a-5p, has been studied for diagnosis and prognostic evaluation. In the therapy aspect, researchers are attempting to explore the role of EVs in the resistance process of NPC treatment, with the aim of clinical translation. Current limitations include biological distribution of EVs and so on. Future research should focus on establishing the standardized production system and more convenient separation and purification techniques for EVs. This review provides a comprehensive overview of the nasopharyngeal carcinoma-related EVs.

## 1. Introduction

Nasopharyngeal carcinoma (NPC) is a type of head and neck malignancy originating from the epithelium of the nasopharynx. It has unique epidemiological features, showing obvious regional clustering, racial differences, and gender differences [1,2]. The etiology of NPC is complex and diverse, involving lifestyle, dietary habits, Epstein–Barr virus (EBV) infection, and genetic susceptibility. Among these, EBV infection is considered a key pathogenic factor, especially in high-incidence areas such as Southeast Asia and southern China [3,4,5]. The treatment strategy of NPC varies with the stage of the disease. According to the treatment guidelines issued by the National Comprehensive Cancer Network (NCCN) [6], radical radiotherapy is preferred for early-stage patients. For patients with locally advanced- and lymph node-metastasis, concurrent chemoradiotherapy (CCRT) is preferred, while for those with recurrence or distant metastasis, multi-drug combination chemotherapy is emphasized. The majority of NPC patients are diagnosed at an advanced stage, and radiotherapy resistance leading to treatment failure, local recurrence, and distant metastasis has become a clinical challenge [7,8]. Therefore, early and accurate diagnosis and prognosis assessment are of great significance for the prevention and treatment of NPC.

Extracellular vesicles (EVs) are lipid bilayer-enclosed particles released by cells into the extracellular environment and are incapable of autonomous replication. The naming conventions for extracellular vesicles are constantly evolving, and there are multiple ways to name them [9] (Table 1). For example, EVs with a diameter below 200 nm are designated as small EVs (sEVs), whereas those exceeding 200 nm are classified as large EVs (lEVs). As critical mediators of intercellular communication, EVs can encapsulate and transfer diverse bioactive molecules, including proteins, nucleic acids, and lipids [10]. Tumor-derived EVs contribute to tumorigenesis, progression, and metastasis by modulating the tumor microenvironment through the delivery of their molecular cargo to recipient cells. The specific biomolecules they carry hold potential as biomarkers for cancer diagnosis and prognosis evaluation. Furthermore, EVs are increasingly being investigated as promising vehicles for tumor-targeted therapeutic delivery [11,12].

The unique properties of EVs render them indispensable in tumor research. NPC, a head and neck malignancy with distinct epidemiological characteristics, lacks a systematic overview of the regulatory roles, underlying molecular mechanisms, and clinical application potential of EVs in its initiation and progression. A comprehensive understanding of the biological features and functional roles of EVs associated with NPC can provide a critical theoretical foundation for elucidating the pathogenesis of NPC and developing novel diagnostic and therapeutic strategies. This review aims to systematically summarize recent breakthroughs in EV-related research, particularly focusing on their role in modulating the NPC tumor microenvironment, as well as their applications in diagnosis and therapy.

## 2. Methods

We searched the PubMed database for articles published between 2020 and 2025 that were related to nasopharyngeal carcinoma-derived extracellular vesicles. The search was organized around five thematic modules by combining “NPC-EVs” with the following terms: tumor microenvironment, biomarkers, treatment resistance, therapeutic strategies, and technological innovation. Studies were screened and included based on their relevance to the overall topic: 17 articles were included for the tumor microenvironment module, 12 for biomarkers, 14 for treatment resistance, 12 for therapeutic strategies, and 6 for technological innovation. We excluded articles that involved NPC-EVs but were not consistent with the overall scope or thematic framework of this review. All included articles were then synthesized and analyzed to provide a qualitative integration of research progress on NPC-EVs, forming the basis of this narrative review.

## 3. Regulation of the Tumor Microenvironment

### 3.1. Mediating Remodeling of the Immune Microenvironment

EVs can not only directly interact with innate immune cells such as macrophages, natural killer (NK) cells, and dendritic cells (DCs) through surface molecules or deliver contents to target them, transmitting regulatory signals, but also regulate the functions of adaptive immune cells such as T cells and B cells through antigen presentation pathways. Through these actions, EVs shape the immunosuppressive or immune-activating phenotypes of the tumor microenvironment, providing protection for tumor escape or inhibiting tumor progression by activating anti-tumor immune responses. The ultimate effect depends on the source of EVs, their contents, and the state of the tumor microenvironment [13,14]. Research has confirmed that NPC-sEVs promote tumor immune escape by inducing mregDCs through carrying galectin-9 (Gal9), reducing the secretion of pro-inflammatory cytokines, and inhibiting T-cells activation [15]. Previous studies have demonstrated through in vivo experiments that, in several cancers, EVs regulate the polarization of macrophages towards an immunosuppressive phenotype through both the direct effect of increased endogenous Programmed Cell Death Ligand 1 (PD-L1) synthesis and the indirect effect of cytokines [16]. However, findings from this research group indicate that although EVs do express PD-L1, their influence on stimulated macrophages is minimal. In contrast, another research team has shown through in vitro experiments that under hypoxic conditions, macrophages upregulate their own PD-L1 expression by phagocytosing NPC-derived EVs expressing PD-L1 (NPC-EVs-PD-L1) [17]. It is agreed that the PD-L1 carried by NPC-EVs can inhibit the proliferation of CD8+T cells both in vivo and in vitro and promote tumor immune escape [17,18]. It is worth considering whether the expression levels of EVs cargo observed in traditional in vitro experiments are consistent with the real levels in vivo. Do the experimental results obtained under normoxic conditions underestimate the immune status in the deep parts of tumors?

**Figure 1:**
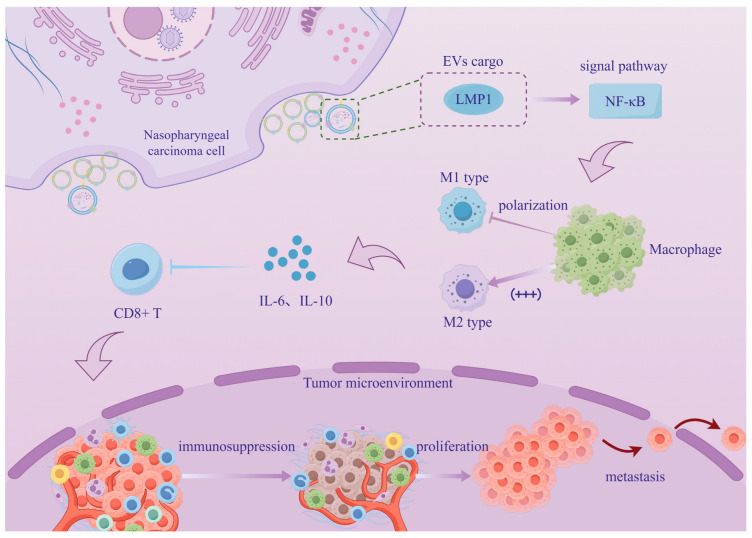
The mechanism by which EVs secreted by EBV-positive NPC cells carry LMP1 and regulate the immune microenvironment. The EVs secreted by EBV-positive nasopharyngeal cancer cells carry LMP1, which is transferred to TAMs. This activates the NF-κB pathway, inducing TAMs to polarize towards the M2 type and secreting cytokines such as IL-6 and IL-10. At the same time, it inhibits the activity of CD8+ T cells, constructing an immunosuppressive tumor microenvironment to promote tumor progression. (By Figdraw 2.0).

According to statistics, the majority of patients with NPC are EBV-positive [19,20], and the latent membrane protein 1 (LMP1) on the surface of EBV+NPC cells not only promotes an increase in the secretion of EVs by interacting with *ALIX* [21] but also ensures that these vesicles carry more toxic cargo to maintain immunosuppression. The study established the protein–protein interaction between LMP1 and *ALIX* through in vitro experiments and demonstrated that targeting *ALIX* could inhibit tumors using humanized mice. However, the clinical data evidence only remains at the level of expression correlation and cannot support the detailed mechanism hypothesis, posing a challenge for clinical translation. Another study demonstrated through in vitro experiments that LMP1 alone could significantly alter the protein cargo and function of EVs in the absence of other viral proteins (Figure 1) [22]. However, some studies suggest that the changes in EVs after EBV infection may be the result of the synergistic action of multiple viral proteins, which are in dispute with this conclusion [23]. Another EV-carried viral protein, BRRF2, can inhibit the cyclic GMP-AMP synthase (cGAS) phase separation in recipient cells, thereby inducing innate immune evasion [24]. Although its mechanism differs from that of host molecules transported by EVs, the result still points to the attenuation of anti-tumor immune surveillance. For instance, the ring finger protein 126 (RNF126) promotes the ubiquitination of Phosphatase and Tension Homologs (PTEN) [25], and miR-193b-3p and Scavenger Receptor Class B Type I (SCARB1) participate in reprogramming macrophages [26,27]. While it is known that cGAS inhibition represents an early innate immune response, the spatio-temporal relationship between this and the reshaping of the immune microenvironment by host molecules remains unclear. Currently, it is unclear whether the vesicles that carry viral proteins and those that carry host molecules belong to the same group of EVs or if NPCs secrete multiple distinct subpopulations of EVs with different functions. While each article emphasizes the decisive role of a particular molecule, they often lack an analysis of the potential co-existence or competitive loading of these cargoes. Studies involving tumor growth in nude mice, which lack T cells, diminish the integrity of conclusions regarding the “immune microenvironment.” As for the regulation of macrophage polarization by EVs, the traditional view is that they promote M2-like polarization [28]. However, the aforementioned co-regulation of M1 and M2 functions by SCARB1 appears to be more biologically plausible, although single-cell sequencing data confirming the state of tumor-associated macrophages (TAMs) is lacking.

By precisely regulating the activation status, functional phenotypes of immune cells, and the signaling pathways within the immune microenvironment, NPC-EVs create a suitable microenvironment for NPC-cells proliferation, invasion, metastasis, and immune escape. This suggests that EVs play a central role in remodeling the NPC immune microenvironment, which makes it possible to become the potential molecular targets for NPC immunotherapy, holding significant translational medical value.

### 3.2. Promoting the Formation of a Pre-Metastatic Niche

During the process of tumor growth and metastasis, new blood vessels are generated to transport nutrients and remove metabolic waste for tumor cells [29]. Current research predominantly focuses on EVs carrying specific functional proteins that target endothelial cells to modulate downstream pathways, thereby influencing angiogenesis and promoting tumor metastasis. Studies have revealed that exosomal High Mobility Group Box 3 (HMGB3) can be internalized by endothelial cells (ECs) to induce angiogenesis [30]. Despite providing a comprehensive chain of evidence encompassing in vitro, in vivo, and clinical data, the precise downstream pathways remain undefined, and the utilized zebrafish model fails to fully recapitulate the long-term progression of tumors. Furthermore, given that HMGB3 is primarily a nuclear protein, the possibility cannot be ruled out that its detected presence in EVs may result from contamination by nuclear debris released during cell apoptosis in experimental settings. Another novel mechanistic insight suggests that EVs contribute to NPC angiogenesis by regulating translation via HS1-related protein X-1 (HAX1) [31]. However, studies have observed no significant correlation between HAX1 and *VEGFA* expression, and potential crosstalk between these two pathways remains unelucidated. In addition, EV-derived LBH has been shown to inhibit NPC angiogenesis by targeting *VEGFA* signaling [32]. If NPC-EVs can both promote and inhibit angiogenesis, the sorting mechanism of these cargos needs to be clarified. Canonically, VEGF activates downstream transcriptional regulation by binding to FLT1 (VEGFR1) and KDR (VEGFR2) [33]. However, recent findings suggest that EV-associated FLT1 functions as a vehicle to transmit activation signals without altering the transcriptional activity of FLT1 within target cells [34]. The dual functionality of FLT1 as both a conventional VEGF receptor and an EVs cargo, compounded by the complexity of the tumor microenvironment, poses significant challenges for clinical translation, particularly regarding the maintenance of equilibrium between these distinct roles.

Fibrosis is one of the characteristics of tumor tissues, which is commonly manifested as increased deposition of proteins in the extracellular matrix (ECM), disordered structure, enhanced post-translational modifications, etc. [35]. Current research suggests that EVs secreted by EBV-positive NPC cells are released into the tumor microenvironment, where they influence the extracellular matrix (ECM) and remodel the tumor microenvironment by targeting adjacent non-tumor cells. These EVs have been shown to induce fibrosis through the YAP1/FAPα axis while concurrently regulating cell–matrix interactions via Secreted Protein, Acidic and Rich in Cysteine (*SPARC*) [36,37]. Given that *YAP/TAZ* transcription factors are established regulators of various secreted matrix proteins [38], it remains to be determined whether a correlation exists between the overexpression of *SPARC* and Yes-associated protein 1 (*YAP1*). Although the study innovatively incorporates the theory of cell competition to explain these mechanisms, the downstream signaling pathways through which *SPARC* orchestrates cell competition remain uncharacterized. Both studies utilize clinical data to demonstrate the potential of their respective molecules as prognostic biomarkers; however, survival analyses are inherently susceptible to numerous confounding factors. In particular, variations in treatment regimens may undermine the accuracy of the established prognostic correlations.

The tumor microenvironment actively mediates the whole process of tumor occurrence, progression, and metastasis through the dynamic interaction of components, which is composed of both cellular- and non-cellular components, including immune cells, tumor-associated fibroblasts, vascular endothelial cells, and extracellular matrix [39]. The current exploration of the mechanism of NPC-derived EVs in the regulation of the tumor microenvironment not only promotes the improvement of the theoretical system of tumor biology, but also deepens the understanding of the mechanism of NPC occurrence and development, immune escape, treatment resistance, and metastasis. At the same time, it provides a multi-dimensional transformation research direction for the clinical diagnosis and treatment of NPC, identifies multiple potential therapeutic targets, and offers theoretical support for further enhancing the precision and safety of clinical treatment.

## 4. Biomarkers for Diagnosis and Prognosis

### 4.1. Diagnostic Biomarkers

Liquid biopsy, owing to its minimally invasive characteristics, enables the detection of circulating nucleic acids, proteins, and other biomarkers in bodily fluids such as plasma and urine, holding significant potential for early cancer diagnosis [40]. Currently, the most commonly used early detection methods for NPC are plasma EBV DNA testing and EBV-VCA-IgA serological testing [41]. With the mechanisms of NPC-EVs in the development of NPC being continuously clarified, liquid-biopsy technology using EVs as biomarkers has come into the public view. For example, the diagnostic marker sEV carbonic anhydrase 1 (CA1), detected by an automated immune–chemiluminescence chip, demonstrated high-sensitivity and specificity in large-scale clinical validation, with an AUC of 0.98. Importantly, its performance was independent of EBV-DNA status and could compensate for the limitations of existing EBV-DNA-based assays [42]. In contrast, studies on another EBV-associated EVs cargo, cyclophilin A (CYPA), provide insufficient evidence for diagnostic specificity in NPC [43]. The discriminatory ability of CYPA between NPC and other diseases was not clearly established, which substantially limits its clinical applicability. To enable large-scale clinical applications, the detection methods for these biomarkers must be both simple and rapid. When EBV-BART13-3p miRNA detected by qRT-PCR is proposed as a diagnostic biomarker for NPC. Whether the detection methodology satisfies the core requirements for large-scale screening warrants further investigation [44]. Although it effectively distinguishes healthy individuals, patients with other head and neck cancers, and asymptomatic EBV carriers, its value in early diagnosis has not been validated, which may restrict its potential for clinical screening applications.

If single EVs cargo biomarkers are insufficient to capture tumor heterogeneity, combinatorial biomarkers can enhance diagnostic robustness through complementary indicators. For instance, multi-marker models based on sEV miRNA expression profiles exhibit strong discriminatory power, demonstrating good resolution across different NPC stages and EBV infection statuses [45]. Such combinatorial approaches not only mitigate detection blind spots caused by the molecular heterogeneity of tumors but also enhance the ability to distinguish clinical difficulties. For example, nano-flow cytometry-based single-particle EVs analysis combined with a five-protein biomarker panel enables accurate discrimination between NPC and nasopharyngitis [46]. However, these combinatorial detection strategies involve relatively complex workflows and impose high demands on sample processing and platform standardization, which currently hinders their clinical implementation. Overall, although these biomarkers have demonstrated associations between EV cargo and NPC through in vitro experiments and clinical data, investigations into causal relationships remain limited, and the potential influence of other diseases on these biomarkers cannot be excluded.

Notably, the integration of data science approaches such as machine learning (random forest algorithms, and so on) is expected to facilitate the integration and high-throughput analysis of EV-based multi-omics biomarkers, thereby opening new avenues for precision medicine [47]. Nevertheless, many existing studies remain at the preliminary screening stage with limited cohort sizes, necessitating further validation to strengthen the robustness of their conclusions. In the future, only through rigorous clinical validation and standardized analytical workflows can EV-related biomarkers be successfully translated from bench to bedside, providing strong support for early screening and precise stratification of NPC.

### 4.2. Prognostic Biomarkers

The prognostic potential of EV-related protein markers has been partially substantiated by clinical evidence. However, considerable heterogeneity in functional roles and expression profiles remains a challenge. Elevated expression of *SPARC* in peritumoral tissues has been identified as a robust predictor of poor prognosis in NPC [37]. However, its clinical utility is constrained by the requirement for adjacent tissue specimens, which introduces practical limitations in sample acquisition and restricts broad applicability. In contrast, *BATF2* functions as a multifunctional biomarker detectable in tissue, serum, and EVs, offering a more convenient detection approach and demonstrating dual potential for treatment-response monitoring and prognostic stratification [48]. Using liquid-biopsy technology as a tool for prognosis assessment can provide information about the possible outcomes of treatment and holds great promise [49]. And its clinical evidence is substantial, suggesting it is closer to clinical translation. However, it still lacks multicenter external validation, and its synergistic prognostic value with traditional clinical indicators remains unclear. Another study verified through in vivo and in vitro experiments and clinical data that EV-miR-30a-5p is associated with survival or metastasis endpoints in NPC patients [50]. Although its evidence chain is more complete compared to *BATF2*, the lack of data stratification by rainfall limits its clinical translation. On the immunological dimension, EV lncRNA TP73-AS1 explains the mechanistic framework of the immunosuppressive microenvironment and poor prognosis, but the clinical evidence remains exploratory and has not yet proven its independent predictive value [51]. Another immune-related marker, PTEN, also faces a lack of clinical evidence, and its value in predicting the efficacy of combined radiotherapy and immunotherapy may be overestimated [52]. Additionally, from the perspective of clinical translation, the nanofluidic technology required for certain EV-based detections has not yet transitioned into routine clinical use. Another marker of radioresistance, collagen α-2 (I) chain, theoretically possesses rapid- and high-throughput potential but faces issues of technological adaptability as well [53]. The surface-enhanced Raman spectroscopy (SERS) chips on which it depends are costly and complex to operate, making them difficult to popularize in primary hospitals. Overall, no EV-related nasopharyngeal carcinoma prognostic markers have been clinically translated as of now. The real gap in the future does not lie in discovering more differential molecules but in establishing reproducible analysis and quantification standards, completing external validation, and proving the benefit to existing clinical decision-making. Additionally, combined detection models may enhance the accuracy and comprehensiveness of prognostic evaluation in NPC.

### 4.3. Detection Technology Innovations

#### 4.3.1. Nano-Engineering

As novel biomarkers are highly promising in the field of oncology, the traditional detection technology of EVs still face bottlenecks such as large sample consumption, poor separation purity, and insufficient detection sensitivity, which is difficult to meet the detection requirements of clinical micro-biological samples. Therefore, research on optimizing tumor-associated EVs detection technology has become the focus of the field. As a powerful tool for gene editing, various Clustered Regularly Interspaced Short Palindromic Repeats-CRISPR associated proteins (CRISPR-Cas) nucleases and their engineered variants have been applied across multiple areas of biological research [54], including enhancing the sensitivity of EVs detection. Current research has integrated CRISPR technology with signal amplification strategies and nanomaterials to address the challenge of detecting low-abundance NPC-EV biomarkers. For instance, combining Cas12a with terminal deoxynucleotidyl transferase or PCR, or pairing Cas14a with rolling circle amplification (RCA), has demonstrated enhanced sensitivity and specificity in EV detection [55,56,57]. These findings are grounded in both in vitro experiments and clinical data; however, a comparative evaluation of the merits and limitations among different CRISPR variants remains elusive, making it difficult to ascertain the optimal subtype for NPC biomarker detection. In theory, such technologies can be readily adapted for detecting EV biomarkers in other tumor types through target substitution, highlighting their strong scalability. Future efforts aimed at promoting large-sample, multi-center validation and advancing the translation into point-of-care testing (POCT) technologies hold promise for eventual clinical implementation.

#### 4.3.2. Microfluidics

While chip technology has driven the innovation of EV analysis, a dichotomy persists between technological sophistication and clinical applicability. Nanofluidic systems excel in isolation fidelity but often remain confined to sample processing without a standalone diagnostic utility [52]. Conversely, physical sensors like SERS have exploration value in terms of signal sensitivity and the holistic molecular “fingerprint” of EVs recognition, yet suffer from poor clinical interpretability of complex spectra [53]. Although integrated immune–chemiluminescence chips provide a standardized, translation-ready workflow, their reliance on single-analyte detection fails to capture EVs heterogeneity [42]. Future breakthroughs necessitate bridging the trade-off between multiparametric profiling and operational simplicity through standardized, clinically aligned integration.

In summary, research on NPC-EVs has advanced biomarker discovery for diagnosis and prognosis, as well as EVs detection technologies, thereby expanding the role of EVs in liquid biopsy and providing new avenues for early detection and precision medicine in NPC. In contrast, current clinical approaches, including invasive tissue biopsy, imaging examinations (CT/MRI/PET-CT), and EBV-related serological tests, remain limited by insufficient sensitivity for early-stage disease, suboptimal prognostic stratification, and inadequate real-time monitoring. With continuous innovations in EVs detection, systematic investigations of EV-associated proteins and genetic biomarkers have deepened, supporting early diagnosis (Table 2), treatment evaluation, and outcome prediction, while also laying a technical foundation for clinical translation. Looking forward, establishing EV-centered dynamic monitoring systems and enabling integrated and standardized detection platforms may facilitate integrated diagnostic–therapeutic strategies, ultimately improving early intervention and precision treatment for NPC.

## 5. Participate in Treatment Resistance

### 5.1. Radiotherapy Resistance

There is a consensus that elucidating the underlying mechanisms of radioresistance is crucial for improving patient prognosis [58]. Accumulating evidence indicates that EVs play an indispensable role in the development of radioresistance in NPC. EVs can encapsulate a variety of functional molecules which are transferred to recipient cells to reprogram their biological behaviors and signaling networks, ultimately shaping the radioresistant phenotype of NPC. On the one hand, NPC-EVs can deliver viral proteins, host proteins, or miRNAs to NPC cells, enhance pro-survival signals, inhibit radiation-induced apoptosis, and suppress pathways such as Akt/mTOR, p38 mitogen-activated protein kinase (p38 MAPK), and Wnt/β-catenin, thereby reducing the sensitivity of NPC cells to radiation [59,60,61]. On the other hand, EVs indirectly upregulate the anti-apoptotic program of NPC cells by inducing immune cells to adopt a pro-tumor inflammatory phenotype, such as the interleukin-17 (IL-17)-related axis [62]. In addition, EVs can promote the transformation of fibroblasts into cancer-associated fibroblasts (CAFs), accompanied by autophagic/metabolic reprogramming, providing energy support for tumor cells through stroma–tumor metabolic coupling and further exacerbating radioresistance [63].

It is noteworthy that EVs can either promote or inhibit apoptosis in NPC cells. For example, EVs carrying CAV1 suppress apoptosis and exacerbate radioresistance in NPC cells, whereas EV-associated miR-142-5p exerts the opposite effect [64,65]. Current studies remain focused on emphasizing the importance of individual mechanisms. What needs to be clarified is how these mechanisms synergize or antagonize each other in the complex in vivo environment, as well as the crosstalk between various mechanisms. At this stage, most molecules should be positioned as mechanism-driven candidate therapeutic targets, and there is still a certain distance from clinical translation. In the future, it is necessary to establish standardized detection methods for EVs and conduct prospective validation of therapeutic targets.

### 5.2. Chemotherapy Resistance

According to the guidelines, the chemotherapy drugs for NPC are recommended to be selected from those based on platinum-based regimens [66]. The researchers are exploring the mechanism of NPC-EVs in chemotherapy resistance, aiming to provide new targets for treatment. As a first-line chemotherapeutic drug for the clinical treatment of NPC [67], the anti-tumor effect of Cisplatin is primarily mediated by inducing tumor-cell apoptosis and other programmed cell death (PCD) pathways [68]. In terms of the mechanism of cisplatin resistance, the apoptosis-ferroptosis regulation mediated by endoplasmic reticulum protein 44 (ERp44), the Protein Kinase B (AKT) activation mediated by miR-106a-5p, and the autophagy regulation mediated by CAF-EVs have all clarified their respective mechanisms through in vitro and in vivo experiments [69,70,71]. However, the synergistic effect of each target has not been explored. Existing studies have proved that EVs transfer signals between resistant cells and sensitive cells through their cargos, thereby targeting drug efflux pumps, cell survival pathways, or cell death programs to regulate chemotherapy sensitivity. On one hand, NPC-EVs can promote chemotherapy resistance. For example, EVs with a high expression of *DDX53* enhance drug efflux ability by upregulating MDR1 expression, inducing NPC cells to become resistant to paclitaxel [72]. On the other hand, NPC-EVs can reverse chemotherapy resistance. For instance, EVs-miR-183-5p reduces drug efflux by targeting and inhibiting P-gp expression, thereby reversing paclitaxel resistance [73]. However, there are differences between in vitro induction of resistant cell lines and clinical natural resistance phenotypes, and the reverse effect of sensitive cell EVs on chemotherapy resistance lacks large clinical sample verification. In the future, challenges such as off-target effects of EVs secretion inhibitors need to be addressed, and specific intervention strategies with high specificity should be developed to promote EVs-related research from mechanism analysis to clinical application.

### 5.3. Immunotherapy Resistance

For patients with advanced and chemoradiotherapy-resistant NPC, immunotherapy is theoretically considered a promising strategy to combat tumor progression [74]. However, the large number of activated Tregs in the TME may affect the efficacy of immunotherapy [75]. Currently, the exploration of how EVs regulate immune-therapy resistance is showing characteristics of innovative mechanisms, but the evidence is mostly from preclinical studies. For instance, γδ-T-Exos have been reported to promote T-cell migration by utilizing the CCR5 ligand secreted by NPC cells, and to resist the immune suppression mediated by TGF-β, thereby enhancing the anti-tumor immune response and potentially improving the benefits of immunotherapy [76]. However, it is necessary to be cautious that such results are mostly based on controlled models and short-term endpoints, and there is a gap from the real resistance environment in the human body. Similarly, some studies have proposed that EVs can transfer circular p-STAT1/2 to macrophages to enhance anti-tumor immunity [77]. The current evidence cannot clarify whether the *STAT* signal transfer mediated by EVs is causal rather than coincidental in the real context of immunotherapy exposure. In summary, EV research provides new mechanism references for NPC immunotherapy, but its current contribution only proposes possible intervention targets rather than clinical tools that can explain and predict resistance. The true value of EVs in NPC immunotherapy resistance needs to be established through causal verification under the pressure of immunotherapy.

NPC patients initially show high-sensitivity to radiotherapy, chemotherapy, and immunotherapy, but most patients later develop treatment resistance leading to therapy failure. Studying the mechanisms of NPC therapy resistance from the perspective of EV-mediated intercellular communication provides a theoretical basis for overcoming treatment failure, preventing recurrence and metastasis, and also offers new targets for treatment.

## 6. Therapeutic Strategies and Technological Advances

### 6.1. As Therapeutic Vehicles

Given the cargo transportation capacity of EVs, they are regarded as an ideal drug-delivery system. More and more studies have attempted to use EVs to deliver a series of therapeutic drugs to specific target tissues [78]. For example, researchers have constructed EVs carriers modified with TMTP1, which can efficiently load ropivacaine and achieve the controllable release of drugs in the acidic tumor microenvironment [79]. Although the TMTP1 modification enhanced tumor targeting, in vivo imaging revealed that some EVs still accumulated in the liver, and the long-term safety issues caused by off-target distribution were not evaluated. Using the carrier that combines Lamp2b protein with iRGD peptide to modify EVs, although it solves the problem of poor targeting of traditional antagomiR delivery and improves the delivery efficiency, also faces the same issues [80,81]. EVs-related tumor vaccines provide a new and efficient feasible solution for developing universal and patient-specific tumor immunotherapy methods, which have important translational application prospects. In animal experiments, they have shown satisfactory safety and stable efficacy [82,83]. EV-related tumor vaccines are a research direction for EVs as therapeutic carriers. However, there is currently no preclinical feasibility verification of EV vaccines for nasopharyngeal carcinoma. It is worth noting that extracellular vesicles derived from mesenchymal stem cells (MSC-Exos) have strong compatibility as drug-delivery carriers for anti-cancer therapy. They not only support multi-mechanism synergistic treatment but also can enhance treatment accuracy through engineered modifications [84]. However, most of the existing studies are preclinical animal experiments, and the long-term safety and organ accumulation toxicity have not been fully verified. And the concentration of exogenous EVs in target tissues after the unmodified EVs in animals are excreted through processes such as bile secretion and renal filtration is extremely low [85]. Therefore, some issues need to be addressed before EVs can be put into clinical use.

### 6.2. Combination Therapy to Enhance Efficacy

Exploring the synergistic effects of EVs with immunotherapy, radiotherapy, and chemotherapy opens new research dimensions for improving NPC treatment outcomes and prognosis. The therapeutic advantage of EV-based combination therapy lies in its ability to precisely target the complex tumor microenvironment in NPC. Current studies suggest that EVs can act as natural nanocarriers, delivering anti-tumor molecules from γδ-T cells and MSC-modified miR-125a to the tumor site [76,84]. At the same time, they can form a synergistic effect when combined with radiotherapy, PD-1 inhibitors, etc. Some studies have regarded them as ideal therapeutic carriers, but other studies have indicated that unmodified MSC-EVs may promote tumor occurrence and inhibit anti-tumor immunity. This means that the therapeutic value of EVs highly depends on the cell source, modification strategy, and tumor microenvironment background. The evidence value obtained under specific experimental conditions needs to be evaluated. The EVs combined therapy strategy proposed under the background of immune regulation ultimately achieves the purpose of increasing the synergistic anti-tumor effect through different mechanisms. For example, EVs are combined with photodynamic therapy mediated by 5-aminolevulinic acid or Stimulator of interferon genes (*STING*) agonists [86,87]. It is necessary to clarify which patients can benefit from such treatment. The imbalance in evidence strength further limits the clinical translation of EV combined strategies. Current studies mainly focus on in vitro experiments and mouse models, which can clearly verify the molecular mechanisms, but the only clinical sample analysis is limited to the correlation studies of markers such as exo-LMP1 in plasma and ALIX in tissues, lacking prospective clinical trials to verify the safety and efficacy of EV combined therapy [21,88].

Over the years, extensive studies have been conducted to determine the value of application of NPC-related EVs, forming a trend to translate basic research findings into applicable intervention strategies. Utilizing the natural advantages of EVs and innovative treatment methods of targeting EVs is (Table 3), on one hand, a new strategy to enhance the effects of existing therapies, expected to overcome the bottlenecks of resistance and side effects associated with current treatments, and on the other hand, provides an ideal technical platform for achieving personalized therapy for NPC.

## 7. Challenges and Future Directions

In recent years, increasing research has focused on the unique biological functions of EVs and their potential applications in malignancy’s treatment. Nevertheless, as a novel biological therapeutic vehicle, EVs face multiple challenges in the clinical translation process. Currently, the standardized production system for EVs has not been established, and the separation methods used by different laboratories are inconsistent, resulting in differences in the characteristics of EVs. There are still differences in the results of EVs biomarkers detected by different methods due to the lack of specific operating procedures. Moreover, because EVs are very sensitive to conditions such as temperature, freeze–thaw cycles, pH, and buffer composition, improper storage of it may lead to alterations in their properties and decrease in their biological activity, or even loss of function, which makes the storage stability of it a key challenge [89,90]. Therefore, it is necessary to establish standardized storage protocols, continue to optimize preservation technologies, and promote the clinical translation process of EVs in future. Now, commonly used isolation techniques for EVs include precipitation, ultrafiltration, size exclusion chromatography (SEC), ultracentrifugation, immunoaffinity capture, and so on. Even though each technology has its own advantages and disadvantages, it is still difficult to balance purity and output, and still there is no technology suitable for clinical large-scale production [91]. Future efforts should focus on optimizing and combining these techniques to enhance the yield and purity of EV isolation.

To date, no completed clinical trials specifically targeting NPC-EVs have been identified. The translation of EV-based strategies from exploratory research to clinical implementation remains constrained by the limited maturity of both technical platforms and the regulatory evidence framework. As the theoretical and biological relevance of NPC-EVs becomes increasingly apparent, key unresolved issues, including variability in pre-analytical handling, heterogeneity in isolation and purification methodologies, and insufficient rigor in EVs characterization and functional attribution, represent critical challenges that must be addressed to enable robust clinical development.

## 8. Conclusions

**Figure 2:**
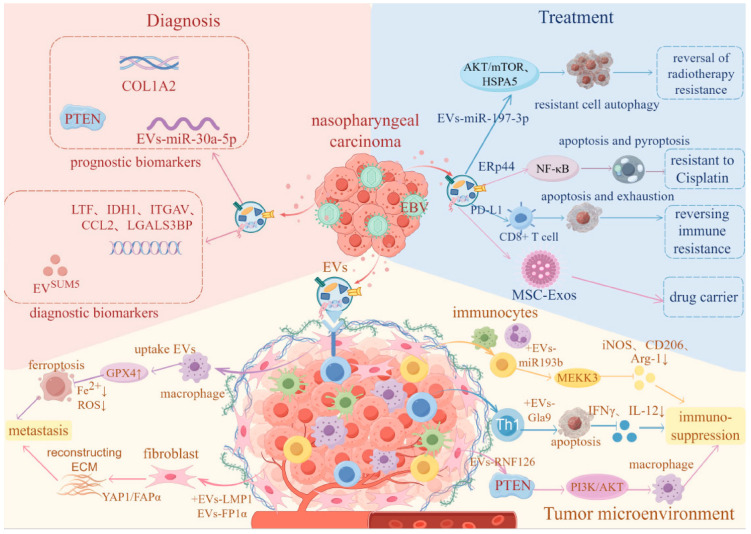
The role of NPC-related EVs in tumor microenvironment, diagnosis, and treatment. The arrows in the figure represent promotion, while the dotted lines at the ends represent inhibition. (By Figdraw 2.0).

In conclusion, this comprehensive review has provided an in-depth analysis of the multifaceted roles of EVs in NPC progression, diagnosis, and therapy (Figure 2). The evidence strongly supports that NPC-derived EVs serve as critical mediators of tumor–host communication, orchestrating various hallmarks of cancer including metastasis, immune evasion, and therapeutic resistance through their cargo of miRNAs, proteins, and other bioactive molecules. Given their potential as valuable tools for enhancing early detection, guiding treatment decisions, and developing targeted therapies in the future, further promotion of their clinical translation will facilitate the development of personalized treatment plans for nasopharyngeal carcinoma patients, ultimately improving patient prognosis and survival.

## Figures and Tables

**Table 1:** EVs subtypes and cargo.

Classification Dimension	EV Subtype	Characteristics	Major Cargo Components
Operational Classification (Size/Density)	Small Extracellular Vesicles (sEVs)	Diameter: Typically <200 nm (often 30–150 nm) Density: 1.13–1.19 g/mL (sucrose gradient)	Proteins: Tetraspanins, TSG101, ALIX, HSPs, transmembrane receptors Nucleic acids: miRNAs, mRNAs, circRNAs, small DNAs Lipids: Phosphatidylserine (PS, inner leaflet), cholesterol, sphingomyelin
	Large Extracellular Vesicles (lEVs)	Diameter: Typically >200 nm (often 200–1000 nm) Density: 1.07–1.13 g/mL (sucrose gradient)	Proteins: Cytoskeletal proteins, membrane-type matrix, integrins, metalloproteinases, annexins, selectins Nucleic acids: rRNAs, genomic DNA (gDNA) fragments, long mRNAs Lipids: Ceramides Phosphatidylserine, phosphatidylcholine Other: Growth factors (VEGF, TGF-β)
	Apoptotic Bodies	Diameter: 1000–5000 nm (1–5 μm) Density: 1.05–1.08 g/mL	Proteins: Histones (H1/3/4, H2A, H2B), caspases (caspase-3/7), apoptotic regulators (p53, Bcl-2 family) Nucleic acids: Genomic DNA, rRNAs
Biogenesis-Based Classification	Exosomes	Diameter: 50–100 nm	Same as sEVs
	Microvesicles	Diameter: 100–1000 nm	Same as lEVs
	Apoptotic bodies	Diameter: 50–5000 nm	Same as Apoptotic Bodies

**Table 2:** EVs-related therapeutic strategies.

Type	Biomarkers	Detection Method	AUC	Advantage	Ref.
protein	sEV CA1	immune–chemiluminescence chip	0.98	not affected by the status of EBV-DNA; high sensitivity and specificity	[42]
protein	CYPA	ELISA	0.84	combine with EBV-VCA-IgA would increase the accuracy of diagnosis, especially when EBV-VCA-IgA is negative	[43]
protein	EV^SUMS^ (LMP1 + LMP2A + PD-L1 + EGFR + EpCAM)	nano-flow cytometry	1	achieved an accuracy rate of 96.3%; sample pretreatment simple	[46]
miRNA	EBV-BART13-3p	qRT-PCR	0.96	diagnostic specificity reached 97%; effectively distinguish healthy individuals, patients with other head and neck cancers	[44]
miRNA	miR-134-5p + miR-205-5p + miR-409-3p	qRT-PCR	0.91	distinguishing patients with different clinical stages and EBV infection status	[45]
DEGs	(LTF, IDH1, ITGAV, CCL2, LGALS3BP)	machine learning	0.99	strong identification power and clinical practicability	[47]

**Table 3:** EV-related Therapeutic Strategies.

EV Type	Mechanism of Action	Therapeutic Effects and Advantages	Application Directions	Ref.
EXOs-miR-197-3p	inhibits AKT/mTOR pathway, reduces NPC cell migration ability; inhibits HSPA5-mediated autophagy, enhances radiation-induced DNA damage and apoptosis.	enhances radio-sensitivity; reduces metastasis risk and chemoresistance; low immunogenicity, suitable for combination therapy.	radiotherapy combination therapy; metastasis inhibition; precision therapy.	[59]
MSC-Exos	regulates MAPK1/PI3K/AKT signaling pathways, inhibits tumor proliferation and angiogenesis, overcomes resistance. loads therapeutic drugs, enables TME-responsive release, improves drug targeting and reduces systemic toxicity.	inhibits resistance-related pathways, reverses radio/chemo-resistance; targeted delivery enhances drug efficacy, reduces normal tissue damage; combines immune regulation and gene therapy, suitable for complex TME.	chemotherapy combination therapy; development of targeted drug delivery systems.	[84]
γδ-T-Exos	preferentially recognizes and binds CD44+/high phenotype radio-resistant CSCs, induces specific apoptosis; retains tumor-killing and T cell activity-promoting effects in immune-suppressive TME.	selectively eliminates radio-resistant CSCs, reduces recurrence risk; enhances systemic anti-tumor immunity; simple preparation, combinable with existing radiotherapy.	radiotherapy combination therapy; CSC-targeted elimination; immunotherapy for immune-suppressive NPC.	[76]
EBV-sEV (LMP1-related)	EBV-encoded LMP1 forms trimeric complex with ALIX and PD-L1, promotes EVs-PD-L1 secretion, mediating immune escape; targeting ALIX inhibits complex formation, reduces sEV PD-L1 release; combined with anti-PD-1 antibody relieves immunosuppression, activates T-cell killing.	targeting ALIX blocks NPC immune escape pathway; ALIX targeting&anti-PD-1 antibody combination therapy effect significantly better than mono-therapy; addresses immune-suppression in EBV+ NPC.	immunotherapy for EBV+ NPC; anti-PD-1 antibody combination therapy; ALIX-targeted drug development.	[21]

## Data Availability

No new data were created or analyzed in this study. Data sharing is not applicable to this article.
